# Impact of *GAUT1* Gene Knockout on Cell Aggregation in *Arabidopsis thaliana* Suspension Culture

**DOI:** 10.3390/biotech14010002

**Published:** 2025-01-02

**Authors:** Tatyana A. Frankevich, Natalya V. Permyakova, Yury V. Sidorchuk, Elena V. Deineko

**Affiliations:** Federal Research Center Institute of Cytology and Genetics, Siberian Branch of Russian Academy of Sciences, pr. Lavrentieva 10, Novosibirsk 630090, Russia; green.faa@yandex.ru (T.A.F.); sidorch@bionet.nsc.ru (Y.V.S.); deineko@bionet.nsc.ru (E.V.D.)

**Keywords:** *A. thaliana*, cell culture, aggregation, genome editing, galacturonosyltransferase, GAUT1

## Abstract

The development of efficient producers of recombinant pharmaceuticals based on plant cell suspension cultures is a pressing challenge in modern applied science. A primary limitation of plant cell cultures is their relatively low yield of the target protein. One strategy to enhance culture productivity involves reducing cell aggregation. In order to minimize cell-to-cell adhesion in culture, we used Cas9 endonuclease to knock out the *GAUT1* gene, which is a key gene of pectin biosynthesis in the genome of *Arabidopsis thaliana*. The resulting knockouts exhibited altered phenotypes and were unable to form viable plants. The suspension cell culture induced from seedlings bearing a homozygous deletion in the *GAUT1* gene displayed darker coloration and an increased number of large aggregates compared to the control. The biomass accumulation rate showed no difference from the control, while the level of recombinant GFP protein accumulation was significantly reduced. Thus, our findings indicate that disruptions in pectin synthesis and the formation of larger aggregates in the suspension cell culture adversely affect the accumulation of the target recombinant protein. Alternative targets should be sought to reduce cell aggregation levels in plant cell cultures through genome editing.

## 1. Introduction

Currently, the majority of biopharmaceutical proteins are not obtained from natural sources but are synthesized as analogs in various expression systems, such as *Escherichia coli*, yeast, mammalian, and plant cell cultures. However, recombinant proteins produced in bacterial cells may not only be contaminated with endotoxins but also lack a range of post-translational modifications characteristic of eukaryotic proteins. The absence of these modifications significantly lowers the quality of bacterial-synthesized recombinant proteins and impacts their biological activity and pharmacokinetics [1,2]. The use of mammalian cells for recombinant protein synthesis is limited by the high cost of cultivation and the potential risk of contamination of the final product with animal pathogens. Recombinant protein synthesis in plant expression systems, particularly in suspension cultures of higher plant cells, avoids most of these drawbacks and combines the ability for eukaryotic-type post-translational modifications with the simplicity and cost-effectiveness of bacterial expression systems [3,4,5]. The success of using plants as bioreactors for the efficient production of high-quality therapeutics has been demonstrated in recent years, particularly during viral epidemics and pandemics (Ebola, SARS-CoV-2), where rapid responses are essential [6,7].

However, plant cells as production systems for biopharmaceutical proteins present certain limitations, one of which is the relatively low yield of recombinant proteins. The causes of low recombinant protein yields are varied, including low transgene expression levels, the displacement of high-yield cells from culture, and high cell aggregation levels in culture. Plant cells grown in suspension are relatively large (50–150 µm) and tend to form aggregates [8]. Aggregates can contain up to 100 cells and may reach sizes of several millimeters depending on the cell line, cultivation conditions, and growth stage [9,10]. Numerous studies have shown that reducing cell aggregation in suspension culture can increase the yield of secondary metabolites [11,12].

Therefore, understanding the factors that influence aggregation in plant cell cultures is critical for optimizing cultivation conditions and maximizing desired outcomes. One of the main causes of aggregation in plant cell suspensions is intercellular adhesion, which is characteristic of plant cells. Aggregation occurs when daughter cells do not separate after division, which is facilitated by extracellular polysaccharides [8]. The polysaccharides constituting cell wall structures are diverse, ranging from simple linear polymers to complex composite molecules [13,14]. Pectins—a broad group of polysaccharides found in cell walls—make up a significant portion of wall polysaccharides and play a role in cell adhesion [15].

The most abundant type of pectin is homogalacturonan (HG), which can be covalently linked to other pectic domains such as rhamnogalacturonan-I or rhamnogalacturonan-II [16]. In the Golgi apparatus, the HG backbone is synthesized in the *cis*- and medial-cisternae by HG: galacturonosyltransferase. *A. thaliana* GALACTURONOSYLTRANSFERASE 1 (GAUT1) was the first identified and biochemically characterized HG galacturonosyltransferase responsible for synthesizing pectin [17]. GAUT1 is clustered in the CAZy GT8 family (https://www.cazy.org/GT8.html accessed on 1 October 2024) together with 14 homologs [17]. GAUT1 is a protein of 673 amino acids with canonical type II transmembrane protein topology, which includes a short N-terminal cytosolic tail, a single transmembrane anchor domain, a structurally undefined linker region often referred to as the stem region, and a catalytic domain facing the Golgi lumen [17]. GAUT1 is cleaved at its N-terminus during maturation and, thus, lacks a membrane anchor itself, but it has been reported to be anchored to the Golgi membrane in a complex with GAUT7 [18]. The GAUT1 enzyme catalyzes the transfer of galacturonic acid from UDP-GalA to HG in the polymerization process [16,17]. The function of this enzyme has been confirmed through biochemical studies [18]. Despite the fact that over time, UDP-GalA-dependent homogalacturonan/galacturonosyltransferase activity was shown for many other representatives of the GAUT family, it is the GAUT1:GAUT7 complex that has the highest activity for de novo HG synthesis [19]. The examples of known mutations in the GAUT family of genes show that the mutations result in a decrease in the content of HG and other pectin components, which, in turn, disrupts the integrity of the cell wall and its mechanical properties. For example, QUA1/GAUT8 is a severely dwarfed mutant that has reduced cell adhesion in expanding leaves and callus tissue and has cell walls with 25% reduced GalA levels [20,21,22]. GAUT10 mutants, showing a decrease in both galacturonic acid and xylose, are observed, which leads to a change in the structure of the cell wall and a decrease in its strength [23]. Thus, based on the fact that GAUT1 is the most active representative of the family, we can assume that disturbances in pectin synthesis caused by mutations in the *GAUT1* gene can lead to the most significant changes in intercellular adhesion, which will lead to a decrease in cell aggregation in suspension cell culture. Reducing culture aggregation may, in turn, lead to an increase in recombinant protein yield.

One of the most promising methods for targeted modifications to the cell genome today is the use of site-specific endonucleases [24], with the Cas9 endonuclease being the most well-known representative. The CRISPR/Cas9 system is based on a guide RNA (sgRNA) containing a 20 bp sequence complementary to the selected genomic target site, along with the Cas9 endonuclease, which induces a double-strand DNA break at this location [25,26,27]. During the repair of this double-strand break, microdeletions and microduplications can occur, potentially leading to gene inactivation. Gene editing with this system has been successfully applied to plants for over 10 years [28,29,30]. The simplicity and ease of use of Cas9 determined its selection for obtaining a knockout of the target *GAUT1* gene.

Thus, the aim of our study is to examine the influence of the *GAUT1* gene on plant cell aggregation and the accumulation level of the recombinant protein in the *Arabidopsis thaliana* suspension culture.

## 2. Materials and Methods

### 2.1. Plant Material

The initial plant material consisted of the transgenic *A. thaliana* (L.) Heynh (Columbia-0 ecotype) homozygous line containing a single copy of the *gfp* gene, which was kindly provided by Zagorskaya A.A. (Institute of Cytology and Genetics SB RAS, Novosibirsk, Russia). The original plant was obtained through agrobacterium-mediated transformation, with random integration of the transgene construct, which was later localized in the genome near the 3′ untranslated regions of the AT4G39600 gene (unpublished data).

### 2.2. Plasmids Carrying Cas9 and Guide RNA

The plasmids pDGE332 (Addgene No. 153241), pDGE334 (Addgene No. 153243) for the intermediate cloning stage, and pDGE347 (Addgene No. 153228) with the Cas9 endonuclease gene under the control of the Arabidopsis RPS5a promoter were kindly provided by J. Stuttmann [31]. As selective markers for plant selection, the constructs included the *FAST* gene, encoding oleosin linked to an RFP-coding gene, resulting in the red fluorescence of the seed coat, and the *Bar* gene, which confers herbicide (phosphinothricin) resistance.

### 2.3. Guide RNA Selection

To inactivate the target gene, two target sites were selected to induce the deletion of a substantial segment of the gene. Guide RNA sequences were identified using the CRISPOR [32] and CRISPR-P v2.0 [33] resources. RNAfold webserver (http://rna.tbi.univie.ac.at/cgi-bin/RNAWebSuite/RNAfold.cgi, accessed on 1 October 2024) was used to analyze the secondary structure. Table 1 presents the oligonucleotide sequences used for assembling the guide RNAs containing the selected guide sequences.

### 2.4. Construction of the Genetic Construct pDGE347_GAUT1

**Figure 1:**
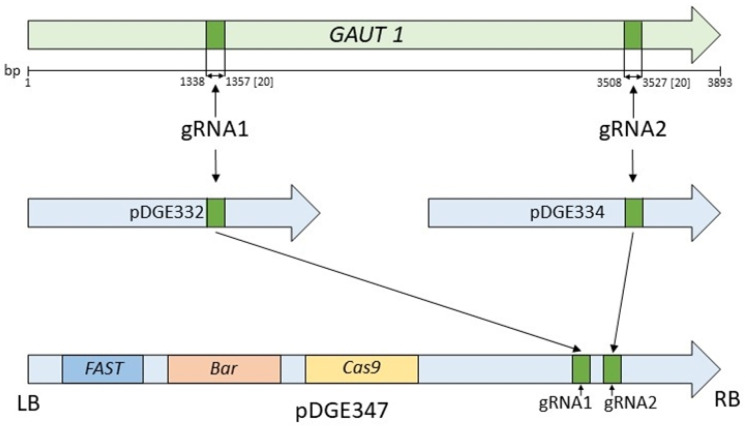
Schematic assembly of the genetic construct for *GAUT1* gene knockout. Designations: *GAUT1*—target gene, indicating target site locations for editing and the distance (in bp) from the starting gene; LB and RB—left and right T-DNA border repeats; gRNA1/gRNA2—target sequences homologous to the *GAUT1* gene target regions; pDGE332/pDGE334—intermediate vectors; pDGE347—the final vector used for plant transformation; *FAST*—gene providing red fluorescent seed coat in transformed seeds; *Bar*—the phosphinothricin N-acetyltransferase gene, conferring resistance to phosphinothricin; and *Cas9*—the Cas9 endonuclease gene.

The selected gRNA sequences were transferred into the pDGE347 plasmid using the intermediate plasmids pDGE332 and pDGE334 for the first and second targets, respectively. The first assembly step involved hybridizing the selected phosphorylated oligonucleotides, followed by their integration into the pDGE332 and pDGE334 plasmids using the Golden Gate method with the BbsI restriction enzyme (New England Biolabs No. R0539S). In the second assembly step, the resulting sgRNAs with the guide sequences were transferred into the pDGE347 plasmid. Integration was performed at the BsaI (New England Biolabs No. R3733S) restriction site using the Golden Gate method. The assembly scheme is shown in Figure 1. To confirm the insertion of the target sequence, DNA from the resulting clones was sequenced using the primers pDGEtest up and pDGEtest lo (Table 1). Sanger sequencing was conducted by the company “Evrogen” (Moscow, Russia).

### 2.5. Delivery of the Genetic Construct into Plants

Agrobacterium-mediated transformation (GV3101 strain of *A. tumefaciens*) was performed using the floral dip method [34], followed by seed selection based on the red fluorescence of the seed coat, detected with a blue-light lamp (Dark Reader Hand Lamp, HL34T, Clare Chemical Research, Dolores, CO, USA).

### 2.6. Analysis of Transformants for GAUT1 Gene Deletion

Seeds selected for seed coat fluorescence were placed in soil and on a sterile MS culture medium [35]. Seeds were sterilized by treating with 4% H_2_O_2_ for 1 min. DNA was extracted from seedlings according to the Kasajima protocol [36], and PCR analysis was performed to confirm the presence of deletion using GAUT1_deltest_Up2 and GAUT1_deltest_Lo2 primers (Table 1). Amplification was carried out with Blitz polymerase (BelbioLab, Moscow, Russia).

### 2.7. Establishment of Suspension Cell Culture

Callus culture was induced from seedlings with deletions in both copies of the *GAUT1* gene by transferring explants to the SH medium [37] (with the addition of 2.4 D—1 mg/L and kinetin—0.1 mg/L, inositol—100 mg/L, 0.8% agar). The callus was then transferred to a liquid SH medium to obtain the suspension culture. For control, callus and suspension cell cultures were also induced from the original Col-0 GFP plants using a similar protocol.

### 2.8. Analysis of Biomass Accumulation and Aggregation in Suspension Culture

To compare the growth characteristics, biomass accumulation analysis was conducted alongside an assessment of aggregation in suspension cultures. Suspensions derived from the cell lines of unedited plants carrying the *gfp* gene were used as controls. For the biomass accumulation assessment, 3 mL of the suspension culture was transferred into 20 mL of liquid SH medium. On days 0, 5, 10, and 15 of cultivation, dry filter papers were weighed, and small and large aggregates were filtered separately using a 1 mm mesh filter. Filters with biomass were dried at room temperature for three days. The mass of the dried biomass was calculated by subtracting the weight of the dry filter paper. The total culture biomass was calculated by summing the mass of small and large aggregates.

### 2.9. Light Microscopy

The visual assessment of aggregates formed in the cell culture was conducted using light microscopy. Samples were stained with trypan blue dye. All images presented were captured using the AxioImager Z1 microscope (Zeiss, Jena, Germany) at 100× magnification with an AxioCam MRm camera.

### 2.10. Analysis of Recombinant GFP Protein and Pectin Levels

To evaluate the accumulation of recombinant protein, GFP levels were measured on the 7th day of cultivation in the suspensions. The amount of GFP protein was determined by measuring fluorescence intensity [38] in extracts obtained from 300 mg of biomass from the suspension culture of each sample. To obtain the extracts, cells were first ground in liquid nitrogen and then centrifuged in PBS buffer. For the normalization of samples based on GFP protein accumulation, the total protein content in the extracts was also quantified using the Bradford assay method [39]. Measurements were conducted using the multimode reader CLARIOstar Plus (BMG LABTECH, Offenburg, Germany).

A commercial Pectin Identification Assay Kit (Megazyme, # K-PECID, Wicklow, Ireland) was used to assess the pectin content. Before the measurement, the samples were lyophilized; a 50 mg portion of lyophilized tissue was used for the measurement. The measurement was carried out on a Bio Rad SmartSpec Plus (Bio Rad, Hercules, CA, USA) spectrophotometer.

### 2.11. Statistical Data Analysis

Statistical analysis of the data was carried out using STATISTICA 10 software. A non-parametric analysis of variance was conducted using the Kruskal–Wallis test, followed by post hoc analysis with Dunn’s test. Differences between groups were considered significant at *p*-value < 0.05 based on pairwise comparisons.

## 3. Results

### 3.1. Analysis of Obtained Plants and Phenotype of T0 Generation

**Figure 2:**
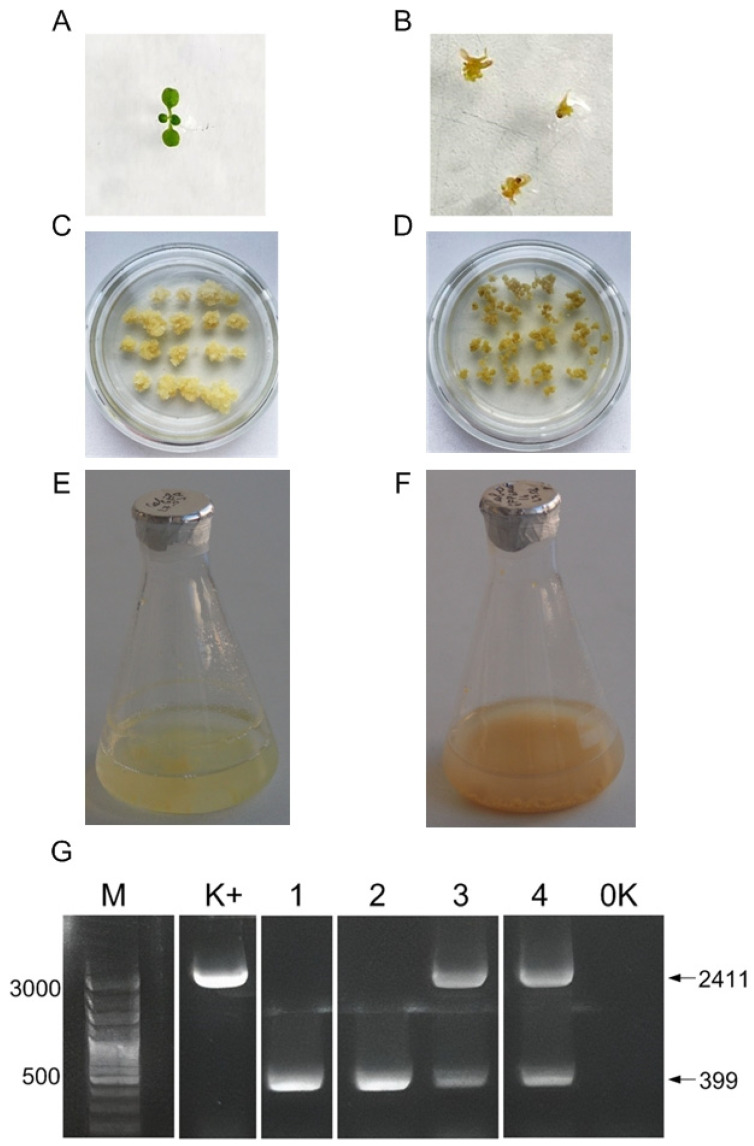
Phenotype and analysis of *A. thaliana* Col-0 GFP and *GAUT1* gene knockout line plants. (**A**)—Seedling of the control Col-0 GFP line; (**B**)—seedlings with *GAUT1* gene knockout, showing morphological abnormalities at early developmental stages; (**C**)—callus culture of Col-0 GFP; (**D**)—callus culture obtained from samples with a deletion in the *GAUT1* gene; (**E**)—suspension culture of Col-0 GFP; (**F**)—culture obtained from samples with a deletion in the *GAUT1* gene; and (**G**)—electrophoresis of the PCR products of plant DNA with the presumed deletion in the *GAUT1* gene in 1.5% agarose gel. Designations: M—DNA marker Step 100 long (Biolabmix, Novosibirsk, Russia), length in bp; K^+^—PCR fragment of the *GAUT1* gene from a control Col-0 GFP line; 1, 2, 3, and 4—PCR fragments of the *GAUT1* gene from a seedling with morphological abnormalities; 0K—negative control; and black arrows indicate PCR fragments of a target gene with or without deletion, with length in bp.

As a result of the conducted agrobacterium-mediated transformation, approximately 200 seeds exhibiting shell fluorescence were obtained. Seeds planted in soil showed very low germination rates—at around 4%. All germinated plants exhibited a normal phenotype. In contrast, seeds placed on a sterile medium demonstrated a higher germination rate of 44%. Among the germinated plants, 39 displayed morphological abnormalities, such as the absence of a well-formed stem and leaves (Figure 2A,B).

The PCR testing of the germinated seedlings with abnormal phenotypes revealed two homozygous and nine heterozygous individuals with a 2012 bp deletion in the *GAUT1* gene. No edited samples were found among the plants grown under non-sterile conditions. An example of PCR analysis for detecting deletions in DNA samples from the germinated seedlings is presented in Figure 2G. The presence of only one short fragment (399 bp) on the electrophoresis gel corresponding to the PCR results from the genomic DNA of the seedlings with morphological abnormalities indicates the homozygous nature of the mutation in the target gene, confirming the presence of deletions in both homologous chromosomes. Morphological abnormalities were observed in all plants with mutations in the GAUT1 gene, both homozygous and heterozygous.

### 3.2. Phenotypic Characteristics of Callus and Suspension Cell Culture with GAUT1 Gene Knockout

Callus and suspension cell cultures were induced from the homozygous *GAUT1* deletion of mutant seedlings. The obtained callus cultures displayed distinct phenotypic differences compared to control callus cultures derived from non-edited seedlings. The *GAUT1*-knockout calluses exhibited a loose structure, which is likely due to disruptions in cell-to-cell adhesion, and were darker in color (Figure 2C,D).

Similarly, suspension cell cultures derived from *GAUT1*-knockout cells showed phenotypic distinctions from the control group (Figure 2E,F). Cultures with mutations in the *GAUT1* gene displayed a darker hue, formed larger aggregates, and demonstrated slower growth rates. The dark-orange coloration is characteristic of cultures experiencing nutrient deficiencies.

**Figure 3:**
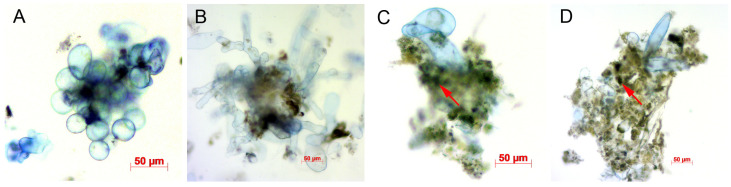
Microphotographs of cells and cell aggregates in cell suspension cultures. Designations: (**A**,**B**)—Col-0 GFP; (**C**,**D**)—*GAUT1* deletion culture; and red arrows indicate clusters of non-polymerized pectin. Scale bar = 50 µm.

A microscopic examination revealed structural changes in the cell aggregates of the *GAUT1*-knockout suspension cultures (Figure 3). In the control culture, cell aggregates consisted of a central cluster of cells bound by cell walls and pectin. However, in *GAUT1*-mutant lines, the cells within the aggregates were smaller, the pectin mass was more voluminous, and cells were positioned around the edges of the aggregates. The typical division center was absent, and the cells did not separate from the aggregates, resulting in a suspension lacking individual cells. The observed mutant phenotype indicated disruptions in cell wall formation during cell division within the aggregate.

### 3.3. Biomass Increase and Aggregation in Suspension Cultures

**Figure 4:**
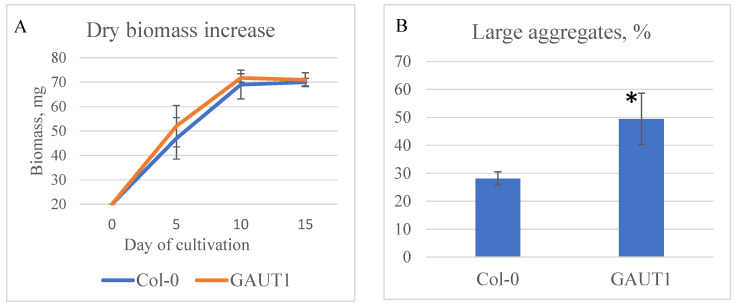
Results of biomass growth analysis (**A**) and aggregation in suspension cell cultures (**B**). Designations: Col-0—suspension culture from Col-0 GFP cell line; GAUT1—suspension culture from the *GAUT1* homozygous deletion cell line. *—Significant differences in the experimental variants from the control line were observed according to the Kruskal–Wallis test at *p* ≤ 0.05.

The results of the comparative analysis of growth characteristics and aggregation between two cell lines, Col-0 GFP- and *GAUT1*-knockout, are presented in Figure 4. Biomass growth analysis indicated no significant difference in biomass accumulation between control and mutant lines according to the Kruskal–Wallis test at *p* ≤ 0.05.

Aggregation analysis, based on comparing the proportion of small (up to 1 mm in diameter) and large aggregates (over 1 mm in diameter) with significant differences as per the Kruskal–Wallis test at *p* ≤ 0.05, demonstrated that *GAUT1* mutants accumulate 21% more large aggregates than the control. Therefore, the obtained mutation did not result in an increased biomass yield compared to the original line but instead led to a higher quantity of large aggregates. These findings are consistent with the microscopic observations of suspension cultures. Apparently, due to disruptions in pectin biosynthesis and normal cell wall formation, cells with a *GAUT1* knockout lack the ability to divide and separate into smaller aggregates.

### 3.4. Analysis of GFP Protein and Pectin Levels

The analysis of the pectin content showed no statistical differences between the control cell line and the cell line with a knockout in the GAUT1 gene (Table 2). The analysis of GFP protein levels in the mutant and control lines (Table 2) revealed that mutant lines accumulated significantly less GFP than the control lines. This observation aligns with the noted changes in aggregation: *GAUT1* knockout mutants accumulate larger aggregates, which subsequently reduce the yield of the recombinant protein.

## 4. Discussion

Cell aggregation in plant suspension cultures is a natural characteristic of plant cells; however, it also has a negative impact on culture productivity. When developing methods to reduce cell aggregation, it is essential to balance the preservation of cellular viability and productivity to ensure there is an efficient system for recombinant protein or secondary metabolite production. Traditionally, physical and chemical methods are used to reduce cell aggregation, including filtration, pipetting, and the addition of small amounts of pectinase [40]. Genome editing offers an alternative by enabling the creation of plant cell cultures with consistently low aggregation without auxiliary interventions. The primary challenge here lies in selecting the appropriate target gene and determining the degree of its modulation—whether through altered activity or complete gene knockout. We chose *GAUT1* as the knockout target because previous studies indicated that mutations in GAUT gene family members could disrupt cell adhesion [20,21,22,23], and the enzyme α-1,4-galacturonosyltransferase 1, encoded by *GAUT1*, is crucial for pectin synthesis [19].

The seeds we obtained after transformation had extremely low germination in the soil, suggesting that even heterozygous *GAUT1* mutations lead to nonviable plants, likely due to early developmental disruptions. Previous work posited that *GAUT1* mutations are lethal in plants [16]. This study is the first to describe the *GAUT1* gene mutant phenotype. Our phenotypic observations of heterozygous and homozygous *GAUT1* mutant seedlings in sterile conditions help explain the lack of *GAUT1* mutations in the existing literature and in known T-DNA mutation libraries, such as SALK [16,41]. The phenotype of the mutant lines we obtained could possibly be explained by the somaclonal variability that arose during transfer to the callus culture; however, the uniformity of the phenotype among all mutant seedlings and cell cultures obtained from them and the absence of such changes in the control line dispute this version. In sterile conditions, our *GAUT1* mutant seedlings exhibited a phenotype consistent with mutants with pectin synthesis disruptions, similar to the growth and morphological abnormalities seen in other GAUT family gene mutants [16,41]. Our measurements showed that the number of pectins in the Col-0 GFP and GAUT1 cell lines remains at the same level; therefore, based on the known data on the function of the GAUT1 enzyme, we believe that the phenotypic changes we observed are caused by the disruption of pectin polymerization. The disruption of HG synthesis caused by the GAUT1 mutation probably stimulates compensatory mechanisms that help plants adapt to changes in the cell wall structure. Data from the literature indicate that a decrease in HG content can enhance the synthesis of rhamnogalacturonan, which maintains the flexibility and extensibility of the cell wall [14].

Beyond growth and developmental differences, we observed color variations between control cells and *GAUT1*-knockout cells, with the mutants exhibiting a darker color in both callus and suspension cultures. Various stress conditions, such as nutrient deficiency, light imbalance, and the accumulation of phenolic compounds or secondary metabolites, are known to cause cell darkening [42,43]. Given that the formation of a fully functional cell wall was impaired in our mutants, we propose that the darker coloration of *GAUT1*-knockout calluses is primarily due to nutrient deficiency. Nutritional stress likely also contributes to the observed reduction in recombinant protein levels in *GAUT1*-knockout cell cultures. Although biomass growth rates remained constant in the mutant line, the increase in large aggregates reduced the number of cells receiving sufficient nutrient access, negatively affecting the productivity of the suspension culture.

Microphotographs of the aggregates also showed that the mass of pectin in the aggregates of the suspension culture with the knockout of the *GAUT1* gene was increased, and the structure of pectin itself was obviously changed. Judging by the fact that there were fewer single cells in such a suspension, the modified pectin had a greater binding ability and contributed to the fact that the cells were more firmly held in aggregates. This increased binding likely resulted from unpolymerized pectin that holds cells in larger aggregates, which fail to break apart and instead continue growing. This hypothesis is based on previous studies on the functions of proteins encoded by these genes [16,17,18,41]. The GAUT1:GAUT7 complex polymerizes HG from galacturonic acid residues [18], and in mutant cells, monosaccharides likely accumulate at high levels, preventing cells from dispersing into smaller aggregates.

It is known that cells in the exponential growth phase undergoing rapid division have a spherical or elliptical shape (50–100 μm), but towards the end of this phase, cells primarily elongate, becoming cylindrical and often reaching up to 200 μm [3]. Larger cells may detach from aggregates and exist independently in cell cultures. The absence of single cells and the predominance of elongated cells in *GAUT1*-knockout culture suggest that most cells are in a late exponential growth phase, where unpolymerized pectin restricts cell division.

Our findings indicate that while pectin biosynthesis genes may appear promising, they are suboptimal targets for knockout if the goal is to reduce cell aggregation in cell cultures. Nevertheless, our work highlights that editing can effectively influence cell aggregation. Alternative targets could include knockouts of genes encoding rhamnogalacturonan 1 and 2 (*RGTX1* and *RGTX2*), which are structural components of the cell wall and play significant roles in cell wall mechanics and intercellular interactions. These findings lay the groundwork for new control strategies aimed at adjusting aggregate size to optimize recombinant protein production and enhance the productivity of plant cell cultures.

## Figures and Tables

**Table 1:** Oligonucleotides used in this study.

Oligonucleotide Name	Nucleotide Sequence (5′-3′)
GAUT1_gRNA1_Forward	ATTGTCTAAAGGAGGGGTCTACTC
GAUT1_gRNA1_Reverse	AAACGAGTAGACCCCTCCTTTAGA
GAUT1_gRNA2_Forward	ATTGGACATTGCCAACTCCAACCA
GAUT1_gRNA2_Reverse	AAACTGGTTGGAGTTGGCAATGTC
GAUT1_deltest_Up2	TTTTTTGGCAGAATCTTGACTGGAG
GAUT1_deltest_Lo2	CAATGGAATGGGAACAACAGAACAT
pDGEtest up	ATAGCAATGACCAGTGCAAACAGTG
pDGEtest lo	CTCTTTTCTCTTAGGTTTACCCGCC
GAUT1_gRNA1_Forward	ATTGTCTAAAGGAGGGGTCTACTC
GAUT1_gRNA1_Reverse	AAACGAGTAGACCCCTCCTTTAGA

**Table 2:** Quantitative analysis of GFP protein content in *A. thaliana* suspension cultures.

Suspension Cell Culture Line	Pectin Level, OE	Total Soluble Protein (TSP), mg/µL	GFP Protein, mg/µL	GFP as % of TSP
Col-0 GFP	0.005	0.40	0.063	16
GAUT1	0.002	1.05	0.06	5.7 *

Note: *—Significant differences in experimental variant values from the control line according to the Kruskal–Wallis test at *p* ≤ 0.05.

## Data Availability

The original contributions presented in this study are included in the article. Further inquiries can be directed to the corresponding author.
